# Reconstruction of anomalous left coronary artery from pulmonary artery in an adult patient: a case report

**DOI:** 10.1186/s13019-019-0866-2

**Published:** 2019-02-26

**Authors:** Kazuki Mori, Hirofumi Anai, Tomoyuki Wada, Takashi Shuto, Shinji Miyamoto

**Affiliations:** 1 Department of Cardiovascular Surgery, Oita University, 1-1 Idaigaoka, Hasama, Yufu, Oita 879-5593 Japan; 2 Clinical Engineering Research Center, Oita University, 1-1 Idaigaoka, Hasama, Yufu, Oita 879-5593 Japan

**Keywords:** ALCAPA, Adult congenital heart disease, Vascular prosthesis, Coronary artery

## Abstract

**Background:**

Anomalous left coronary artery from the pulmonary artery is a congenital heart disease in which myocardial ischemia occurs within 1 year of birth. Adults have been reported to survive owing to the development of collateral perfusion from right coronary artery. In these cases, however, revascularization is necessary to prevent sudden cardiac death.

**Case presentation:**

A 62-year-old female gradually started experiencing dyspnea during exercise. Coronary computed tomography revealed that the left coronary artery arose from the main pulmonary artery. The patient was subsequently diagnosed with adult-type, anomalous left coronary artery from the pulmonary artery. She underwent a surgery, in which a 6-mm vascular prosthesis was passed through the main pulmonary artery to anastomose the left coronary artery and ascending aorta.

**Conclusion:**

Our reconstructive technique using vascular prosthesis is effective for anomalous left coronary artery from pulmonary artery in adults.

## Background

Anomalous left coronary artery from the pulmonary artery (ALCAPA; also called Bland–White–Garland syndrome) is a rare, congenital, cardiac anomaly caused by a coronary arterial malformation during the early stages of heart development. Unless this anomaly is properly treated during infancy, ALCAPA usually leads to death within the first year of life due to myocardial ischemia in the left coronary artery (LCA) region and acute ischemic mitral regurgitation. However, adults have been reported to survive without symptoms due to the development of a collateral circulation from the right coronary artery (RCA) to the LCA. However, even in such cases, surgery is essential to prevent heart failure and sudden cardiac death. In this study, we report the surgical correction of ALCAPA in a 62-year-old female.

## Case presentation

A 62-year-old female with no illnesses in the past and who ran marathons in her 30s started experiencing difficulty in breathing during exercise since her 60s. Previous electrocardiograms obtained in her 40s showed some abnormalities, which were unknown to us. On admission to our hospital, an electrocardiogram obtained during the treadmill test revealed a complete left bundle branch block. Echocardiography showed an enlarged RCA (10 mm) and a vessel with blood flow into the pulmonary artery (PA). Left ventricular ejection fraction was 60% and mild mitral regurgitation was noted. Coronary computed tomography (CT) revealed that the LCA arose from the dorsal side of the PA and that both the coronary arteries were markedly dilated and tortuous. On performing cardiac catheterization, the contrast medium was observed to flow from the RCA into the PA via the LCA; the pulmonary/systemic blood flow ratio was 1.4 and pulmonary artery pressure (systolic/diastolic/mean) was 39/19/28 mmHg. Adenosine-loading myocardial scintigraphy revealed ischemia in the left anterior descending branch.

**Fig. 1:**
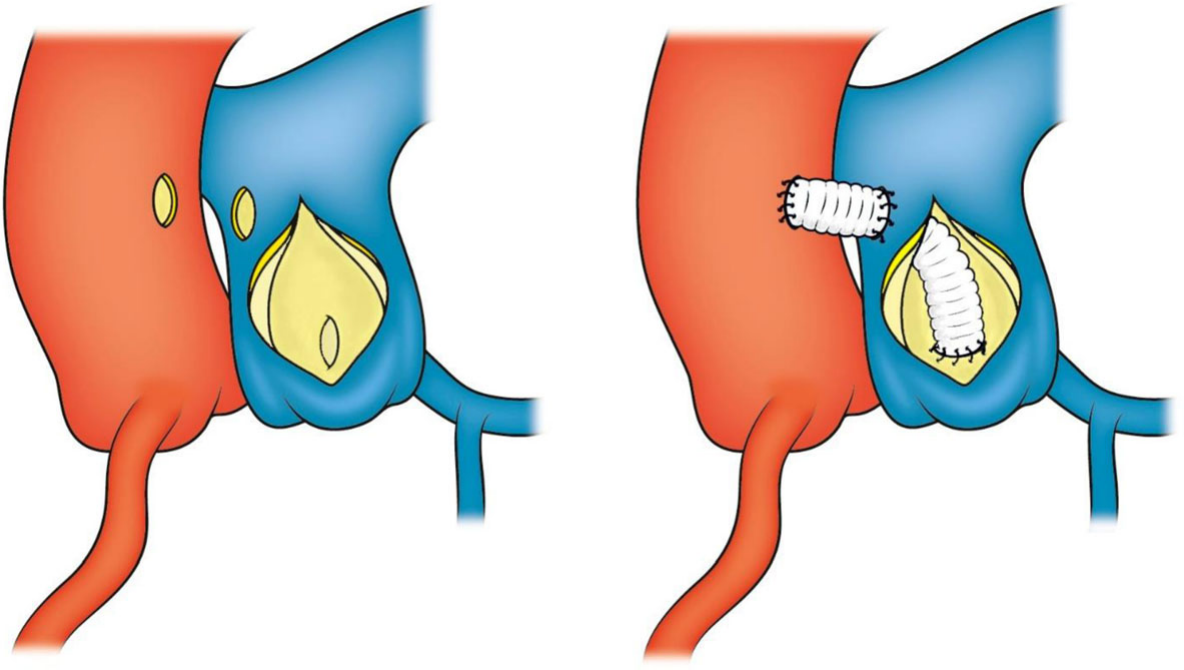
Schematic diagram of the surgical procedure. The ostium of the anomalous left coronary artery was located on the dorsal side of the pulmonary artery. A 6-mm vascular prosthesis was anastomosed end-to-end at the ostium of the anomalous left coronary artery. The vascular prosthesis was then passed through the pulmonary arterial wall and was anastomosed end-to-side with the ascending aorta

**Fig. 2:**
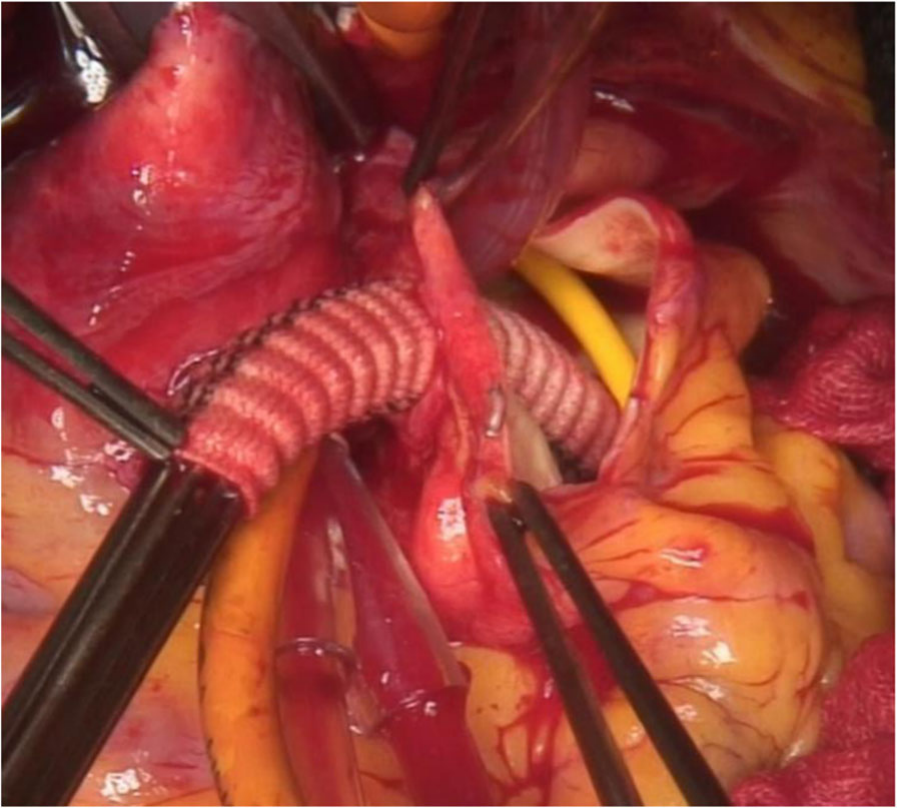
Intraoperative figure. Intraoperative view of the vascular prosthesis anastomosed end-to-end at the ostium of the left coronary artery and passed through the main pulmonary arterial wall

Subsequently, surgery was performed using median sternotomy. A cardiopulmonary bypass was established from the superior and inferior vena cava to the ascending aorta. The patient was then cooled to 32 °C. The PA was longitudinally incised, following which the ostium of the LCA was located. Cardiac arrest was induced using an antegrade injection of the cardioplegic solution from the ascending aorta. Following injection, the surgeon occluded the retrograde flow in the LCA with his index finger to prevent leakage of cardioplegic solution from the RCA. A 6-mm Gelsoft™ Plus (Terumo, Tokyo, Japan) was anastomosed end-to-end using a 5–0 Polypropylene suture at the ostium of the LCA in the PA. A small hole was then made in the PA by incising the aortic side. Subsequently, the anastomosed vascular prosthesis was then passed through the hole in the PA wall. The PA wall and protruding part of the vascular prosthesis were sutured using a 5–0 Polypropylene suture. Thereafter, the vascular prosthesis was anastomosed end-to-side to the ascending aorta using a 4–0 Polypropylene suture. After de-clamping of the ascending aorta, the PA incision was closed [Figs. 1 and 2]. The surgery was terminated after assessing the cardiac function as good.

Postoperative hemodynamics, including blood pressure and cardiac output were stable and no ischemic change was observed. After resuming meals postoperatively, the patient was administered oral aspirin (100 mg) and warfarin (till the prothrombin time-international normalized ratio became around 1.5). Postoperative echocardiography showed no inflow from the coronary artery into the PA, and no jet suggestive of supravalvular pulmonary artery stenosis due to the implanted vascular prosthesis was observed. Coronary CT confirmed the patency of the vascular prosthesis. Subsequently, the patient was transferred to another hospital for rehabilitation on the 10th postoperative day.

**Fig. 3:**
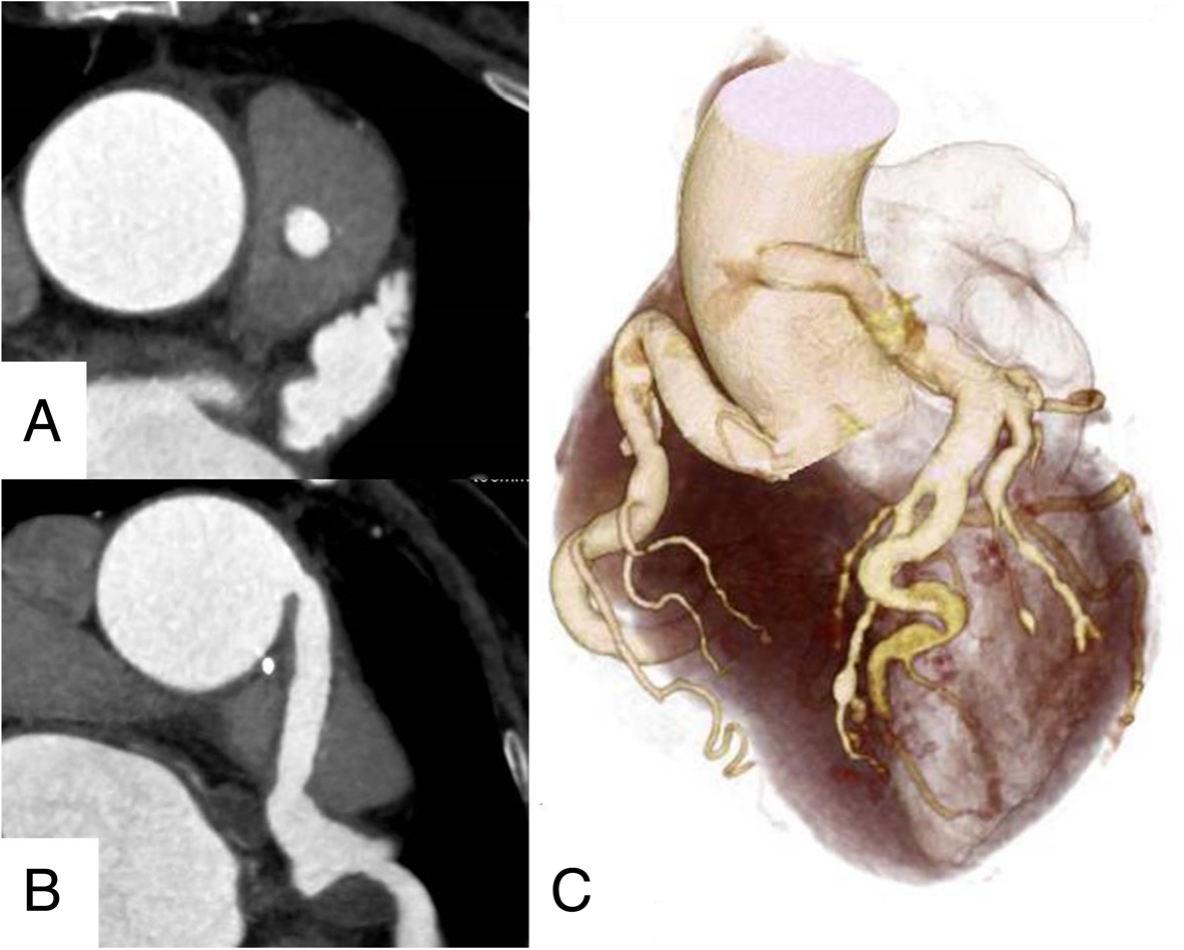
Post-operative computed tomographic image. **a**, **b** Postoperative computed tomography scan revealed the patency of the vascular prosthesis implanted in the main pulmonary artery at 2 year. The main pulmonary artery is also sufficiently patent. **c** 3D computed tomographic image revealed the vascular prosthesis penetrated through the main pulmonary artery

**Fig. 4:**
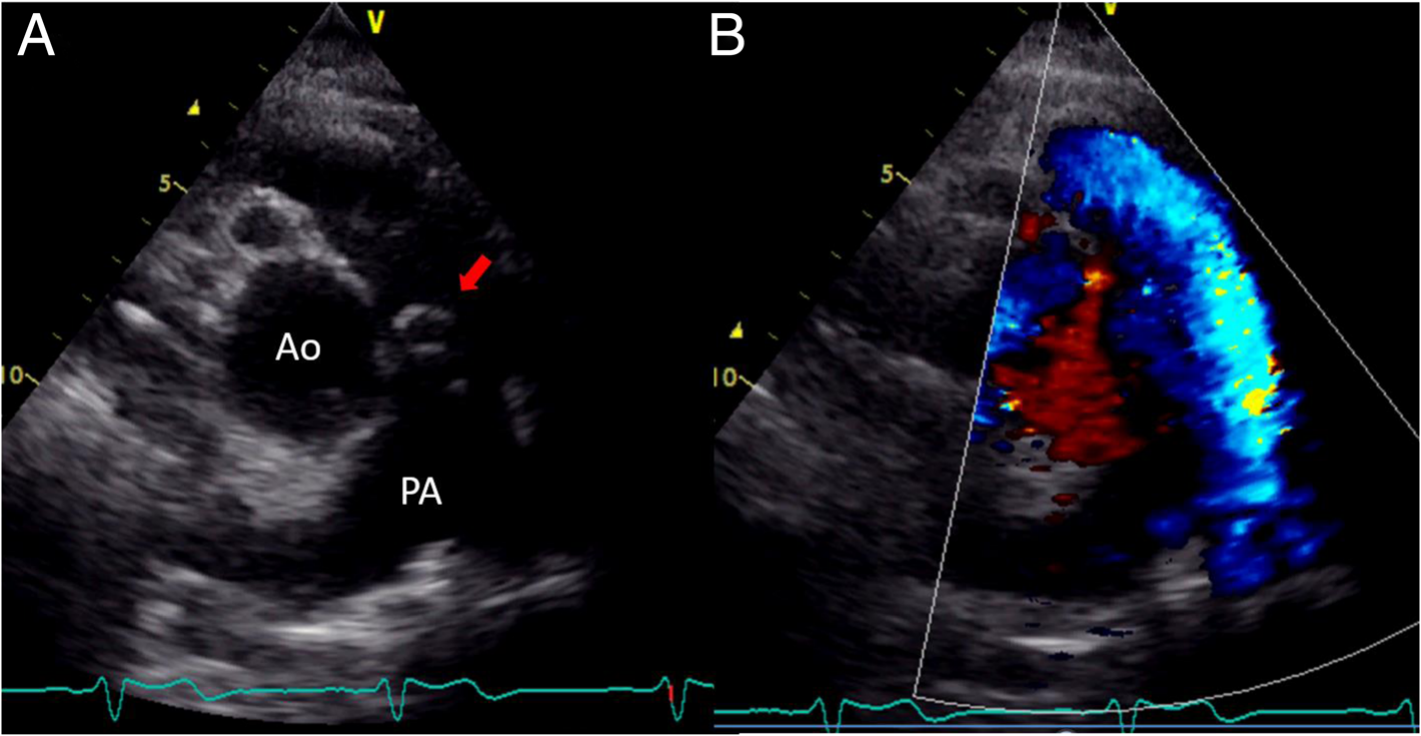
Post-operative echocardiogram. **a** Post-operative echocardiogram revealed the patency of the vascular prosthesis. **b** No stenotic blood flow was revealed in the color Doppler. Ao: aorta, PA; pulmonary artery, allow; vascular prosthesis

Coronary CT obtained after 2 postoperative years showed a patent graft that was anastomosed to the coronary artery [Fig. 3]; moreover, no stenosis of the PA was observed at echocardiography [Fig. 4]. The patient did not admit symptoms and did not need any re-intervention for 2 years.

## Discussion and conclusions

ALCAPA is a rare congenital, coronary artery malformation with a reported incidence of 1/300,000 live births, accounting to 0.25–0.5% of all diagnosed cases of congenital heart diseases [1]. In ALCAPA, the LCA that originates from the PA causes myocardial ischemia in the LCA region. The neonatal closure of the ductus arteriosus and physiological reduction in the pulmonary vascular resistance results in early occurring left-ventricular dysfunction and ischemic mitral regurgitation. As a result, over 90% of neonatal patients diagnosed with ALCAPA die within 1 year of birth. However, because of the development of a collateral circulation from the RCA, a small number of such patients survive until adulthood without suffering from myocardial ischemia. Nevertheless, even in such asymptomatic cases, sudden death may occur due to coronary steal; therefore, immediate surgical intervention is necessary after diagnosis [2]. In the present case, exercise load electrocardiogram and adenosine-loading myocardial scintigraphy indicated the presence of myocardial ischemia.

In ALCAPA, it is necessary to revascularize the arterial blood to improve the perfusion of the LCA. The most physiologically compatible method of revascularization is to directly transplant the origin of the LCA to the aorta; however, in adults the mobility of the tissues surrounding the LCA are poor, making transplantation difficult. Several tunneling method had been reported and the Takeuchi repair has been proposed. In Takeuchi repair method, a tunnel in the wall of the main PA is constructed from the LCA to the ascending aorta [3]. Although several adult patients have been treated using this procedure have been reported, stenosis within the tunnel or just above the pulmonary valve is reported as the long-term complications [4]. Therefore, alternate procedures involving aortic or PA wall flaps to extend the LCA and those for performing coronary artery bypass surgery with LCA ligation have been reported [5–9]. As the number of cases of adults with ALCAPA is less, no standard surgical approach has yet been defined. We selected our method for two main reasons.

Firstly, all these procedures require skillful techniques to prevent the bending or occlusion of the blood flow path leading to the LCA including the tunnel. Secondly, the size mismatch between the graft and dilated coronary artery is of concern when performing coronary artery bypass. In our procedure, stenosis in the PA due to the presence of the vascular prosthesis and thrombosis in the vascular prosthesis can become problematic. However, in the present case, we assumed that the 6 mm vascular prosthesis will not affect PA stenosis because the diameter of the main PA is sufficiently large (25 mm). Moreover, postoperative echocardiography revealed no findings suggesting stenosis in the main PA. Thrombus formation in the vascular prosthesis was prevented using aspirin and warfarin. However, long-term outcomes of this technique remain unclear, to the best of our knowledge, there are no reports regarding reconstruction using vascular prosthesis in patients with ALCAPA. Since, in our case, there has been no problem in postoperative course for 2 years, we consider that reconstruction of ALCAPA using vascular prosthesis is useful.

## Abbreviations

ALCAPA: anomalous left coronary artery from pulmonary artery
CT: computed tomography
LCA: left coronary artery
PA: pulmonary artery
RCA: right coronary artery
